# A Physical Mechanism and Global Quantification of Breast Cancer

**DOI:** 10.1371/journal.pone.0157422

**Published:** 2016-07-13

**Authors:** Chong Yu, Jin Wang

**Affiliations:** 1 State Key Laboratory of Electroanalytical Chemistry/Changchun Institute of Applied Chemistry, Chinese Academy of Sciences/Changchun, Jilin 130022, China; 2 College of Physics/Jilin University, Changchun, Jilin 130012, China; 3 Department of Chemistry, Physics & Applied Mathematics/State University of New York at Stony Brook/Stony Brook, NY 11794-3400, United States of America; CNR, ITALY

## Abstract

Initiation and progression of cancer depend on many factors. Those on the genetic level are often considered crucial. To gain insight into the physical mechanisms of breast cancer, we construct a gene regulatory network (GRN) which reflects both genetic and environmental aspects of breast cancer. The construction of the GRN is based on available experimental data. Three basins of attraction, representing the normal, premalignant and cancer states respectively, were found on the phenotypic landscape. The progression of breast cancer can be seen as switching transitions between different state basins. We quantified the stabilities and kinetic paths of the three state basins to uncover the biological process of breast cancer formation. The gene expression levels at each state were obtained, which can be tested directly in experiments. Furthermore, by performing global sensitivity analysis on the landscape topography, six key genes (HER2, MDM2, TP53, BRCA1, ATM, CDK2) and four regulations (HER2⊣TP53, CDK2⊣BRCA1, ATM→MDM2, TP53→ATM) were identified as being critical for breast cancer. Interestingly, HER2 and MDM2 are the most popular targets for treating breast cancer. BRCA1 and TP53 are the most important oncogene of breast cancer and tumor suppressor gene, respectively. This further validates the feasibility of our model and the reliability of our prediction results. The regulation ATM→MDM2 has been extensive studied on DNA damage but not on breast cancer. We notice the importance of ATM→MDM2 on breast cancer. Previous studies of breast cancer have often focused on individual genes and the anti-cancer drugs are mainly used to target the individual genes. Our results show that the network-based strategy is more effective on treating breast cancer. The landscape approach serves as a new strategy for analyzing breast cancer on both the genetic and epigenetic levels and can help on designing network based medicine for breast cancer.

## Introduction

Cancer is one of the most dangerous and fatal disease at present. The global cancer mortality increased by 8% from 7.6 million in 2008 to 8.2 million in 2013 [1]. Breast cancer is the most commonly diagnosed cancer and the primary cause of deaths from cancer in women, accounting for over 23% of all the cancer cases and about 14% of the cancer-related deaths [2].

With the high mortality rates of cancer, early diagnosis will be vital for breast cancer survival. Many reports showed that if detected and treated promptly, 5-year relative survival is over 93% for localized breast cancer. In contrast, 5-year survival will drop to less than 24%, if the cancer has spread to other organs [3]. And there will be much suffering for patients during therapy in this period. Therefore, it is of great importance to diagnose cancer in time for immediate treatment. However, people often go for therapy when they have already developed late-stage cancer. Clinical observations have shown that traditional methods are not efficient at early diagnosis of breast cancer.

There has been considerable studies suggesting that cancer is a disease caused by gene mutations [4, 5]. Accumulation of mutations has been regarded as the essential characteristic of the six hallmarks of cancer [6]. On the other hand, more recently, some researchers propose that cancer is a particular natural cell state associated with complex molecular networks [7–9]. Molecular networks in mammalian cells are important for controlling cell proliferation, differentiation and apoptosis. Some approaches based on micro-array data aiming to predict metabolic cancer genes receive certain attentions [10–13]. The transformation from normal cells to cancer cells can be caused by changes in these molecular networks which contribute to cancer cell autonomy [14, 15]. In other words, if there is something wrong with the regulation of genes or transduction of signals in the system, some cells do not necessarily follow the instructions normal cells are subject to and cancerization may start. Great effort has been made to reveal the mechanisms of cancerization. However, it is still challenging to describe these complex biological processes systematically and quantitatively.

The determination of receptor targets is the major obstacle in drug design. The potential causes and phenotypes of breast cancer are often varied. This has made the design of drugs against breast cancer much more complex and it is difficult to formulate a clear strategy for effective treatment of breast cancer. Computational models and experiments which aim to rationalize and overcome the experimental bottleneck are widely used on drug target prediction [16, 17]. In general, the drugs targeting on the single gene or the protein can be specific and have less side-effects on normal tissues, but they are often only suitable for early stage of cancer. The drugs applied to malignant stage such as anti-angiogenesis therapy often damage the normal tissue at the same time.

To address the above issues, we constructed a gene regulatory network (GRN) of breast cancer and developed a landscape model to uncover the mechanisms of breast cancer. Then we provide a method to detect premalignant stage for early diagnose. Furthermore, we develop a network-medicine based drug designing method which not only focuses on individual genes but also involves adjusting key regulation strengths among the genes. The network-medicine based drug designing method based our landscape approach can be used to design the treatment for specific stage of breast cancer with less side-effects and damage on other normal tissues. The method is based on the quantification and understanding of the underlying mechanisms of cancerization. We also explore the present clinical experiments to validate the feasibility of our prediction.

The data used for the GRN construction is obtained from experimental literatures. 15 crucial genes associated with breast cancer are included in the network. The nonlinear dynamical interactions in the GRN can generate various stable states with biological functions. In practice, some states may be relatively easy to detect; others may be not. From the landscape topography, the biological functions of these states can be quantified clearly. The kinetic paths between different states illustrate the mechanisms of cancerization.

Through analyzing the stabilities and biological characteristics, three stable fixed-point attractors can be identified and quantified, representing the normal, premalignant and cancer state, respectively. In the normal state, the cell growth, arresting and apoptosis obey the rules they normally follow. The premalignant state is a condition in which the cells grow with some abnormal features resembling certain cancer characteristics. In the cancer state, cell growth becomes uncontrollable and eventually spread to other organs of the body. The premalignant state is between the normal state and the cancer state. It may progress into the cancer state, or it may return to the normal state if proper treatment is taken in time. However, the premalignant state is difficult to detect with traditional examinations. This non-obvious biological state can be obtained through quantifying the underlying landscape of the GRN. This can help with early diagnosis and prevention of breast cancer.

To simulate the internal and external stimuli in cellular environments, we changed the strength of regulations in the GRN. This leads to variations of the landscape topography and kinetic paths. The trends of these changes are consistent with experimental results. Through global sensitivity analysis, genes and regulations critical for breast cancer can be identified. Among the results, HER2 and MDM2 are the most effective drug targets for breast cancer treatment; BRCA1 and TP53 are the most crucial oncogenes and tumor suppressor genes of breast cancer, respectively. This further confirms the robustness of our model. Among the key regulations we found, ATM→MDM2 received more attentions in the experimental community on DNA damage. Here we found this regulation is important for regulations of breast cancer. Validations of the key regulations will help us to design network-medicine and this will lead to more targeted treatments. The network-medicine based drug designing approach can be suitable for treatment of breast cancer with less side-effects and damage on other normal tissues.

This study presents a novel but simple method which systematically and quantitatively reveals the formation of breast cancer. It also offers new insight into early diagnosis of cancer and the design of polygenic anti-cancer agents. The curing strategy for breast cancer can be improved by adjusting relevant polygenic regulations in the network effectively.

## Results and Discussions

### The Gene Regulatory Network wiring of breast cancer

**Fig 1:**
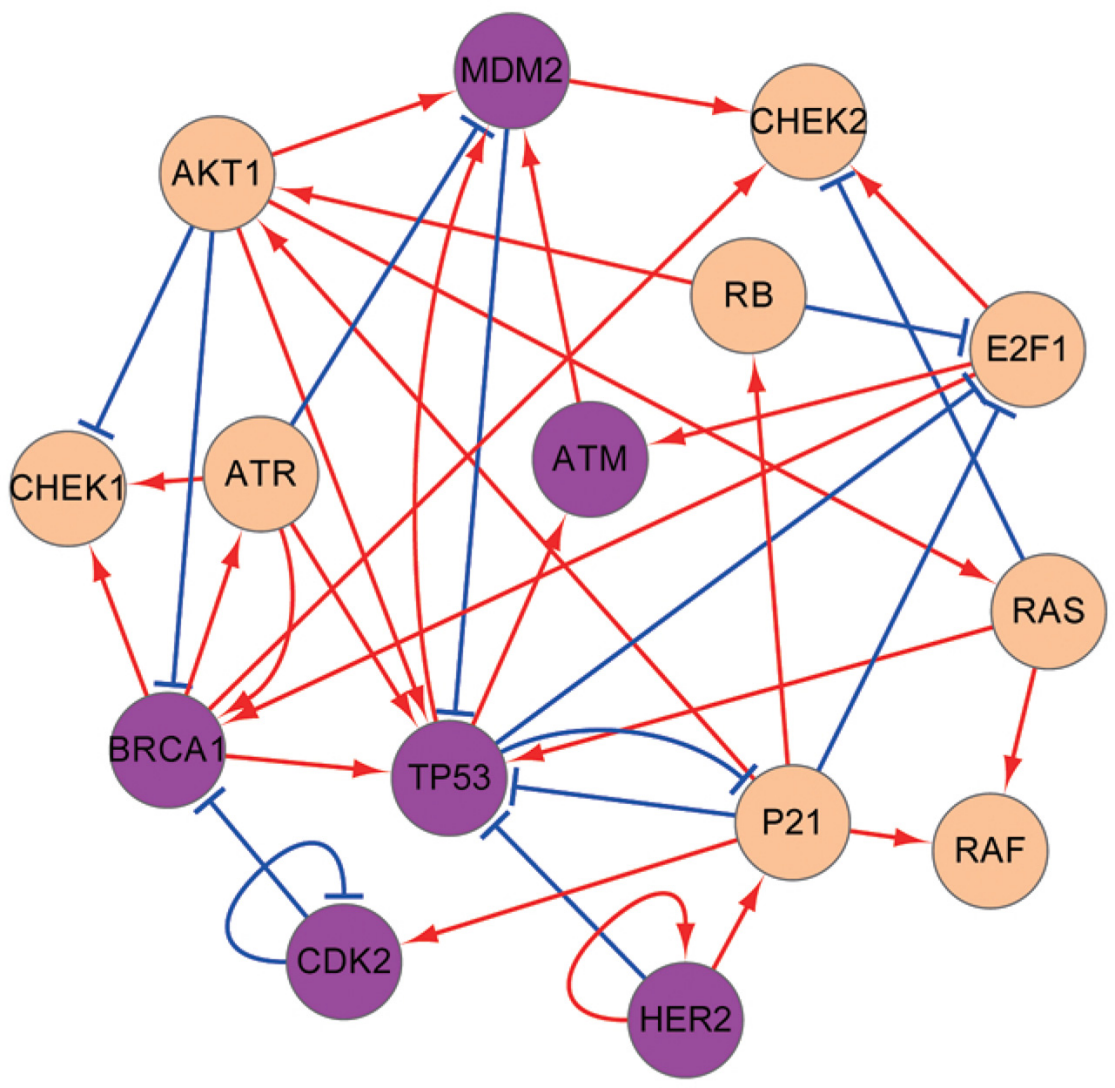
The diagram for the GRN of breast cancer. The GRN contains 15 nodes (genes) and 39 edges (26 activation interactions and 13 repression interactions). The arrows means activation interactions and the short bars represent repression interactions.

In order to uncover a reliable GRN for breast cancer, we searched for the data from experimental literatures (see Supplementary S1 Table). Fig 1 shows the 15 genes in the GRN which are crucial for breast cancer. Magenta nodes represent important genes identified from global sensitivity analysis (see later for details). Each regulation in the GRN is obtained from related experimental data and biological pathways. This GRN contains the following genes: oncogenes as BRCA1, MDM2, RAS, HER2; tumor suppressor genes as TP53, P21, RB; kinases as CHEK1, CHEK2, AKT1, CDK2, RAF, essential to the maintenance of cell cycle regulations; the transcription factor E2F1; and ATM, ATR, which play critical roles in early signal transduction through cell-cycle checkpoints (shown in Supplementary S2 Table). This GRN also includes the main functional pathways: BRCA1-TP53 for signaling tumor suppression [18]; ATR-CHEK1 vital to the maintenance of genome stability [19]; TP53-P21-RB related to apoptosis [20]; MDM2-TP53 which modulates p53-dependent metabolic regulation [21]; ATM-MDM2-TP53 involved in DNA damage response [22]; ATR-CHEK1 and ATR-TP53 necessary for oxidative stress [23, 24]; ATR-TP53-P21 which responds to DNA damage and pathogenesis of cancers [25]; RB-E2F1 regulating the initiation of DNA replication [26]; HER2-TP53 related to the regulation of telomerase [27]; HER2-P21-AKT whose connection to patients’ survival has been evaluated by immunohistochemical staining [28]; RAS-RAF which is a highly conserved pathway critical in normal cell function maintenance.

Once we have the network wiring, we can mathematically describe the dynamics of GRN in the following way. The temporal evolution of the gene network dynamics is determined by the driving force involving gene regulations.

$$\begin{array}{c} \frac{dX_{i}}{dt}=F_{i}=-K_{i}\times X_{i}+\sum_{j=1}^{m_{1}}\frac{a_{j}\times {\left(X_{j}\right)}^{n}}{S^{n}+{\left(X_{j}\right)}^{n}}+\sum_{j=1}^{m_{2}}\frac{b_{j}\times S^{n}}{S^{n}+{\left(X_{j}\right)}^{n}}. \end{array}$$ (1)

The $\frac{dX_{i}}{dt}$ represents the individual gene expression changes with respect to time. The driving force for the gene expression changes consists of three parts. They represent regulations among genes: self-degradation, activation and repression, respectively. *K* is the self-degradation constant; *a* is the activation constant; *b* is the repression constant. *X*_*i*_ denotes the expression level of gene *i*. The Hill function is characterized by two constants. *S* represents the “threshold” of the sigmoid function, at which point the function has value 1/2. *n* is the Hill coefficient which depicts the steepness of the sigmoid function representing the cooperatives of the transcription factor regulatory binding to the genes [29]. In our model, we set *S* = 0.5 and *n* = 3. The activation and repression terms in the equation represent the regulatory relationship from other genes to a certain gene. For gene *i*, the sum in the second (third) term is over the nodes which have activation (repression) interactions with node *i*, where *m*_1_ (*m*_2_) is the number of nodes that activate (repress) node *i*. Here, in Eq (1), *i* = 1, 2, …, 15. So there are 15 equations to describe the network. For simplifications, we assume that the weight of each edge is “1”.

### Potential Landscape and Global Optimal Paths of the GRN of breast cancer

**Fig 2:**
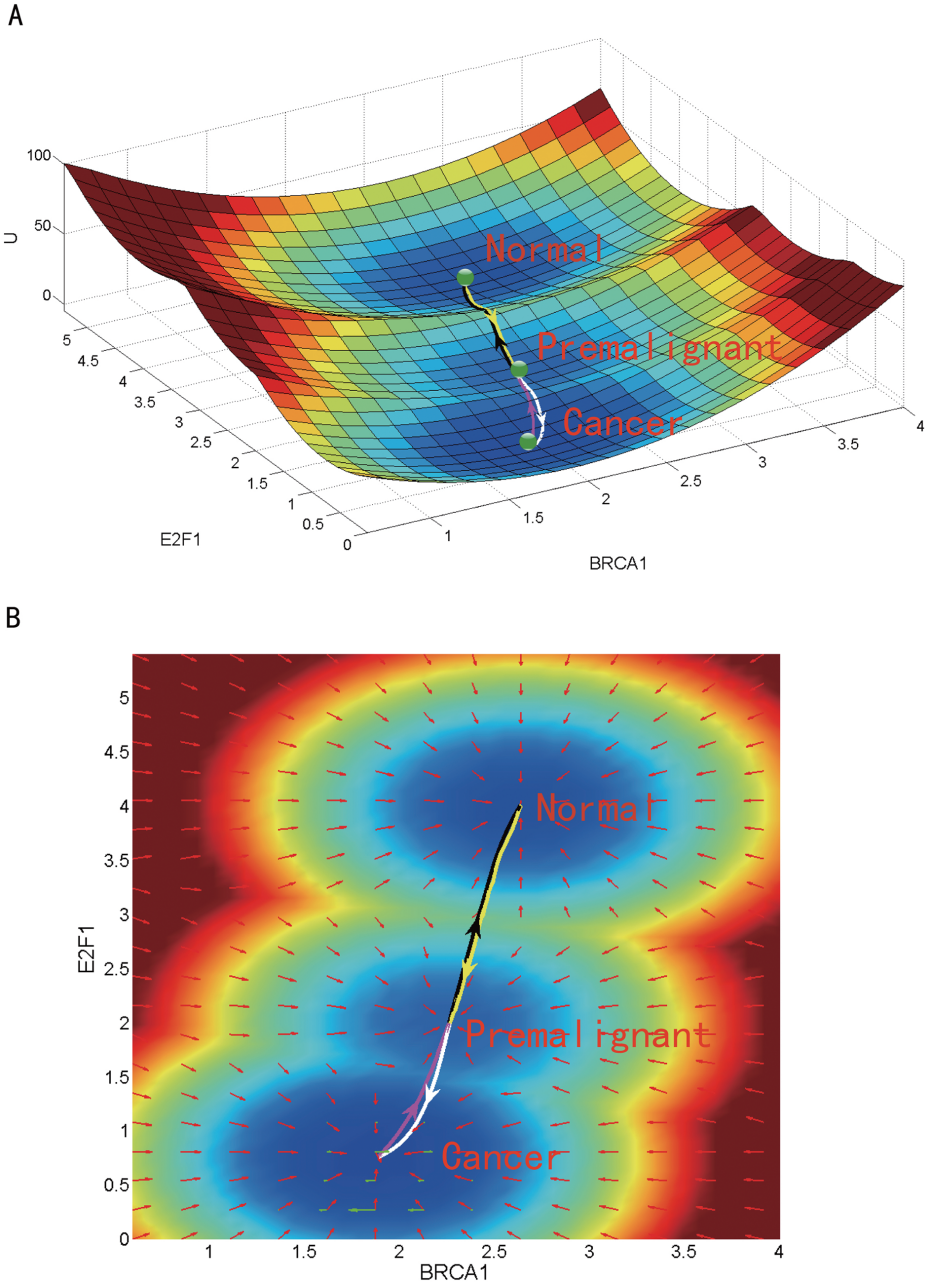
The tristable landscape of the breast cancer GRN. The parameters are set as follows: the degradation constant *K* = 1, the activation constant *a* = 1, the repression constant *b* = 2, and the diffusion coefficient *D* = 0.03. (A) The three dimensional landscape and dominant kinetic paths. (B) The corresponding two dimensional landscape of the GRN. The lines in white, magenta, yellow and black represent respectively the dominant kinetic path from the premalignant to the cancer state, from the cancer to the premalignant state, from the premalignant to the normal state, and from the normal to the premalignant state. Red arrows and green arrows represent the negative gradient of the potential landscape and the probability curl flux force, respectively.

Cancer formation is a polygenic process. A nonlinear mathematical model was adopted to describe the interactions of the breast cancer GRN. The deterministic dynamics of this GRN can be described by Eq (1). Based on the self-consistent mean field approximation, we got the steady-state probability distribution *P*_*ss*_ of the breast cancer GRN. The dimensionless potential landscape *U* was then obtained using the definition *U* = −ln *P*_*ss*_ [30, 31]. It is difficult to visualize the landscape in a 15-dimensional space. Thus we projected the landscape onto a 2-dimensional subspace spanned by the expression levels of BRCA1 (an oncogene of breast cancer) and E2F1 (an biomarker of breast cancer) as shown in Fig 2.

**Fig 3:**
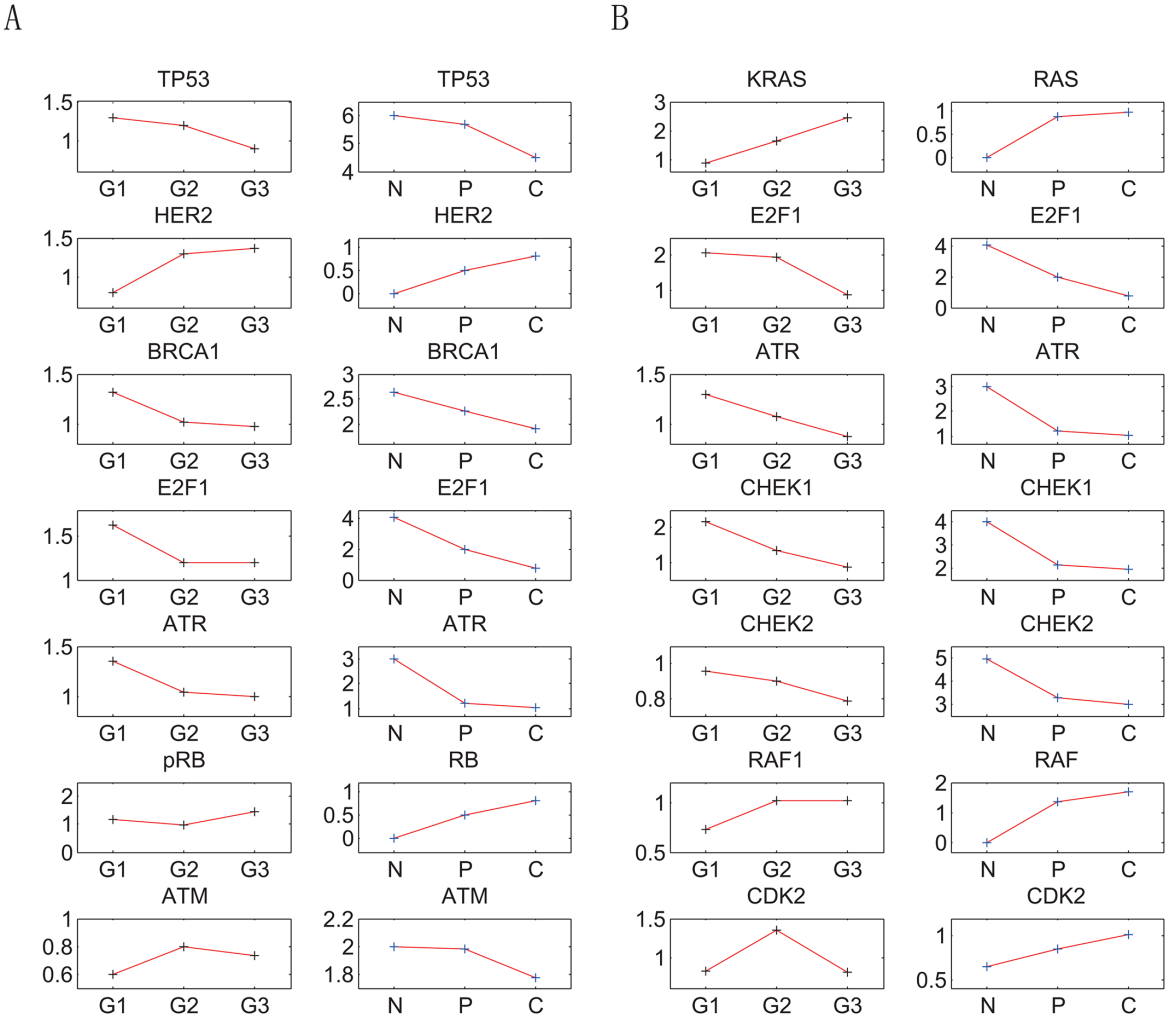
The comparison of experimental results of gene expression levels and our theoretical predictions. The y-axis of left column of the each of the two figures represents fold changes of the gene expression levels, the y-axis of right column of the each of the two figures represents our theoretical predictions. The label G1, G2 and G3 in the x-axis represent grade1, grade2 and grade3 grades of breast cancer. The label N, P and C in the x-axis represent normal state, premalignant state and cancer state. (A)Shows the data of carcinoma in situ. (B)Shows the data of metastatic carcinoma.

There are three attractor basins on the landscape. They are identified respectively as the normal, premalignant and cancer state, according to their gene expression levels and biological functions. The simulated gene expression levels in each attractor state agree qualitatively with the clinical data of the corresponding cell state. The numerical results show that, in the cancer state, MDM2, AKT1, CDK2, P21, HER2, RB, RAF and RAS have high expression levels, while ATR, TP53, ATM, BRCA1, CHEK1, CHEK2 and E2F1 have low expression levels. In the normal state, in contrast with the cancer state, ATR, TP53, ATM, BRCA1, CHEK1, CHEK2 and E2F1 have high expression levels, while MDM2, AKT1, CDK2, P21, HER2, RB, RAF and RAS have low expression levels. The simulation results of the premalignant state are in between those of the cancer and normal state (see Supplementary S3 Table). These numerical results are consistent with available experimental data. As demonstrated by experimental results (see Supplementary S3 Table), MDM2, AKT1, CDK2, P21, HER2, RB, RAF and RAS are often over-expressed in breast cancer, accompanied by the loss of gene functions of ATR, TP53, ATM, BRCA1, CHEK1, CHEK2 and E2F1. The consistency between the modeling results and the experimental data in the gene expression levels support the identification of the three attractors on the landscape with the corresponding biological cell states. Fig 3 provides a quantitative comparison of the trend of cancer grades as cancer progresses from Microarray data (GSE14548) and our landscape calculations. The grading, in pathology, is a measure of cell anaplasia. The higher the grade is, the more poorly differentiated and more dangerous it is. The states characterized by the attractors in our landscape calculations quantified the stages of breast cancer. When the attractor is in premalignant state, it corresponds to the very low grade level. When the attractor is in cancer state, it corresponds to the very high grade level. In Fig 3, the expression levels of thirteen genes have been calculated at different stages and compared with the Microarray data. Ten of them are consistent with the variation trends of the Microarray data. Our landscape calculations are consistent with the trends of the cancer grades. This further validates the identification of these the three attractors with the respective cell states.

Moreover, as can be seen from Fig 2(A), the attractor basin of the normal state is fairly deep. It means the normal state is pretty stable against fluctuations. The cell is not likely to leave the normal state attractor under weak fluctuations. Yet when genetic mutations and environmental fluctuations become large enough, the cell has a higher chance to overcome the barrier between the normal and premalignant state attractors and thus transform into premalignant state. The attractor basin of the premalignant state, located between the normal and the cancer state attractors, is relatively shallow. Hence, it is relatively easy for the cell in the premalignant state to turn into the normal and the cancer states. The attractor basin of the cancer state is rather deep, indicating that the cancer state is quite stable and difficult to escape from. These explain the premalignant state is difficult to detect and cancer is difficult to cure. This illustrates the biological functions of the three attractor cell states.

The kinetic paths among normal, premalignant and cancer states were quantified by the method developed earlier [31]. The landscape contour in 2-dimensions is shown in Fig 2(B). It can be seen that the optimal path from normal to premalignant state (black line) and that from premalignant to normal state (yellow line) are almost identical and reversible with each other, while the optimal path from premalignant to cancer state (white line reversible) and that from cancer to premalignant state (magenta line) are slightly separated. This can be explained that the driving force of the gene regulation system can be decomposed into a gradient of the potential and a curl flux force [30]. When the curl flux force is strong, the kinetic path will deviate from the steepest descent path determined by the gradient of the potential, so that the two kinetic paths connecting the cancer and premalignant states are not exactly along the gradient of the potential. Therefore the two paths become separated. The fluxes in the basins of normal state and premalignant state are relatively weak. Thus the two kinetic paths are almost along the gradient of the potential, making them almost identical.

### Genetic and Non-Genetic dependence of landscape topography of the GRN

**Fig 4:**
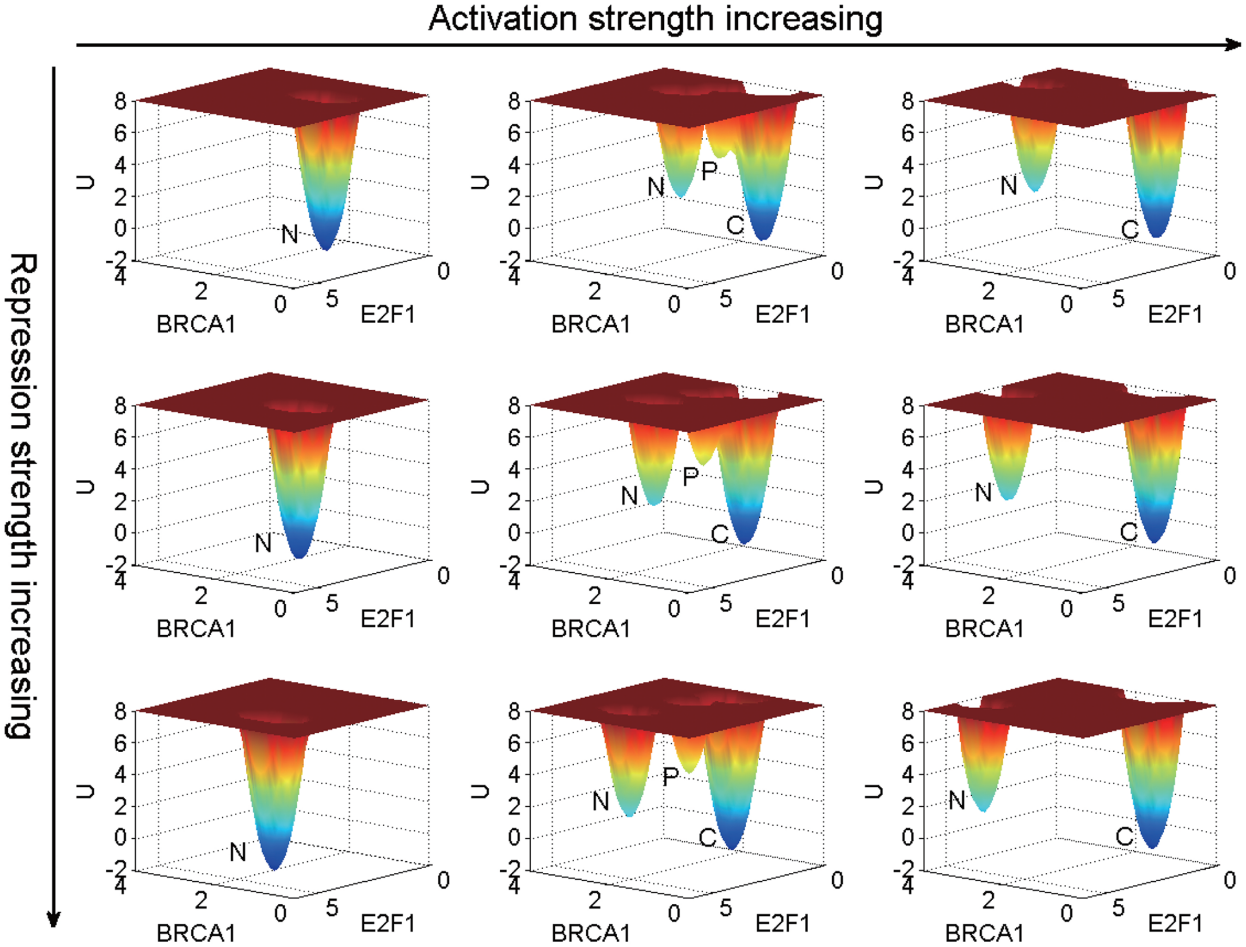
The landscape topography with different activation and repression strengths. From left to right the activation parameter *a* is 0.5, 1 and 1.5, respectively. From top to bottom the repression parameter *b* is 1, 1.5 and 2, respectively. The label *N*, *P*, *C* represent, respectively, the normal, premalignant and cancer state. The diffusion constant *D* is 0.03.

To check the dependence of the landscape topography on both genetic and non-genetic changes, we varied the overall regulation strength parameters *a* for activation and *b* for repression. According to the numerical results, we find that *a* governs the qualitative characteristics of the landscape topography (*a* < 1 monostable, *a* = 1 tristable, *a* > 1 bistable), while *b* determines the variation of barrier heights. The landscape of breast cancer GRN in the space of BRCA1 and E2F1 gene expression levels is shown in Fig 4.

As seen from Fig 4, the GRN has a monostable state (normal state), tristable state (normal, premalignant and cancer states) and bistable state (normal and cancer states) as *a* increases from 0.5, 1 to 1.5. When the activation strength is under the standard level (indicated by *a* = 1), the premalignant and cancer states disappear and only the normal state survives. In contrast, when the activation strength is higher than the standard level, the premalignant state disappears and the cancer state is dominant. In other words, higher activation strengths are often associated with the higher risks of cancer. Higher activation strengths are related to higher gene expression levels (shown in Supplementary S6 Table) and metabolic rates. There is a close connection between the gene expression levels and metabolic rates. Global suppression of the gene expression levels serves as a major cause of the metabolic rate supression in all systems of hypometabolism [32]. A lower metabolic rate will have lower risk of cancer. Some large animals with low specific metabolic rates, such as elephants and whales, do not suffer from cancer, as low metabolic rate can alter cancer cells and reduce the risk of cancer [33]. A possible explanation is that higher metabolic rate and gene expression level result in higher oxidative stress and mutational rates, which could be linked to a higher incidence of cancer. Instances mentioned above indicate that higher metabolic rate and gene expression level may be related to higher risk of cancer; lower metabolic rate and gene expression level may suppress the formation of cancer. This statement is supported and illustrated by the qualitative change of the landscape topography with respect to the activation strength shown in Fig 4.

**Fig 5:**
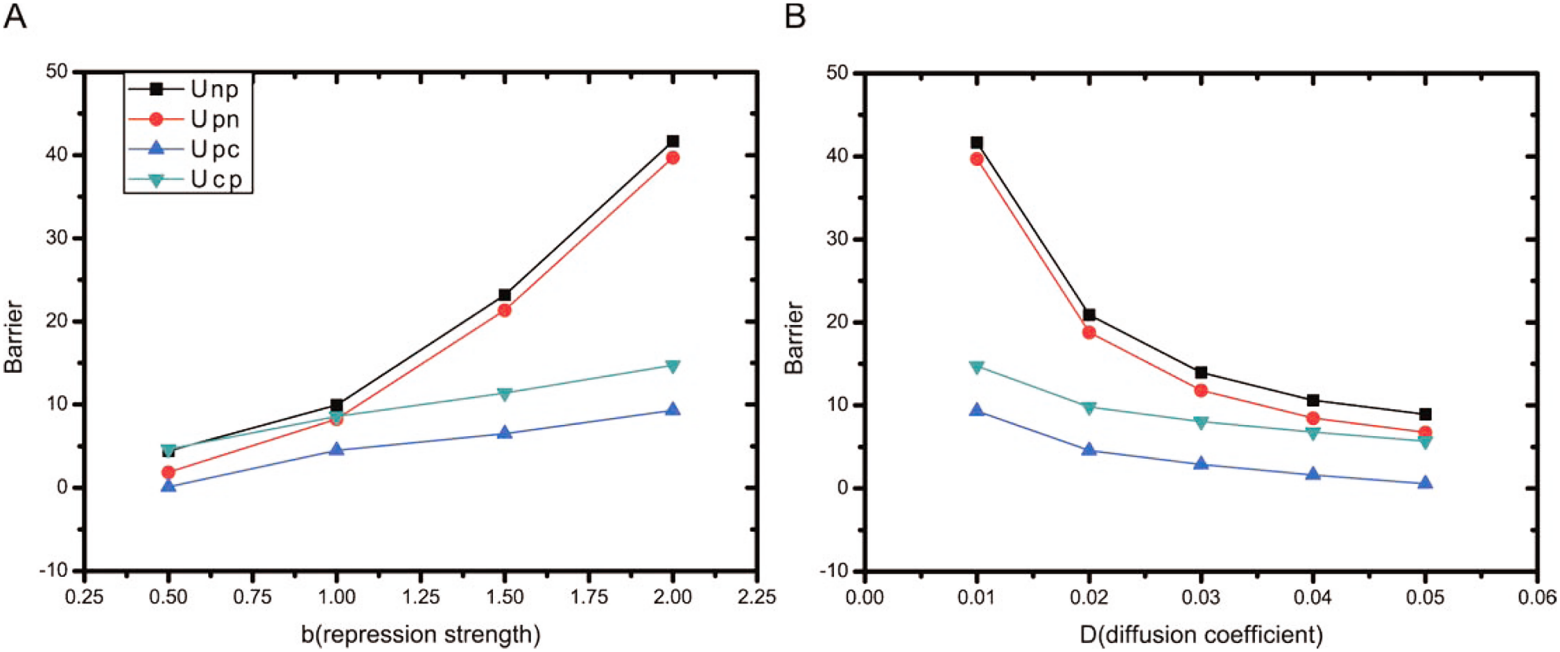
The barrier height results when the parameters changed. (A) shows the variations of barrier heights with the repression strength *b*, for fixed noise level *D* = 0.03 and activation strength *a* = 1. (B) shows the variations of barrier heights with the noise level *D*, for fixed repression strength *b* = 2 and activation strength *a* = 1. *U*_*np*_ (*U*_*pn*_) denotes the potential difference between the saddle point separating the normal attractor and the premalignant attractor and the local minimum in the normal (premalignant) attractor. *U*_*pc*_ (*U*_*cp*_) represents the potential difference between the saddle point separating the premalignant attractor and the cancer attractor and the local minimum in the premalignant (cancer) attractor.

The changes of the barrier heights in the tristable state (activation strength *a* = 1) with respect to the repression strength *b* and diffusion constant *D* are shown in Fig 5. As we can see from Fig 5(A), when the repression strength *b* increases, the barrier heights increase. This is related to a characteristic best seen in Fig 4. In Fig 5(B), when the diffusion constant *D* (quantifying the noise level or fluctuation strength) increases, the barrier heights decrease. More specifically, the barrier heights between the normal and premalignant states decrease more significantly than those between the premalignant and cancer states when *D* increases. That means, relatively speaking, it is easier to go from the normal state to the cancer state than the reverse process. That means the risk of cancer rises with noise level. In the cellular environment, the fluctuations including DNA damage, incorrect signal transduction, protein concentration change, pH variation, oxygen consumption etc.. If the fluctuations is within a controllable range, the immune system will use the natural killer cells to wipe out the cancerous cells. The TP53-P21 dependent pathway in our GRN is in response of killing the cells lacking p53 activation [34]. It means the system could be held to be normal or reversed from premalignant to normal state. But if the fluctuations are largely beyond the control of the immune system, the cancerous cells will start to spread. Then the barrier between premalignant and cancer state has been overcome and cancer state emerges. This illustrates when the fluctuation (the diffusion coefficient *D* in Fig 5(B)) increased, the barrier heights go down. Thus it is easier to go from normal to cancer state. An increased fluctuations can be viewed as an essential compensatory mechanism of cancerization.

### Finding key genes and regulations of breast cancer from global sensitivity analysis of landscape topography

**Fig 6:**
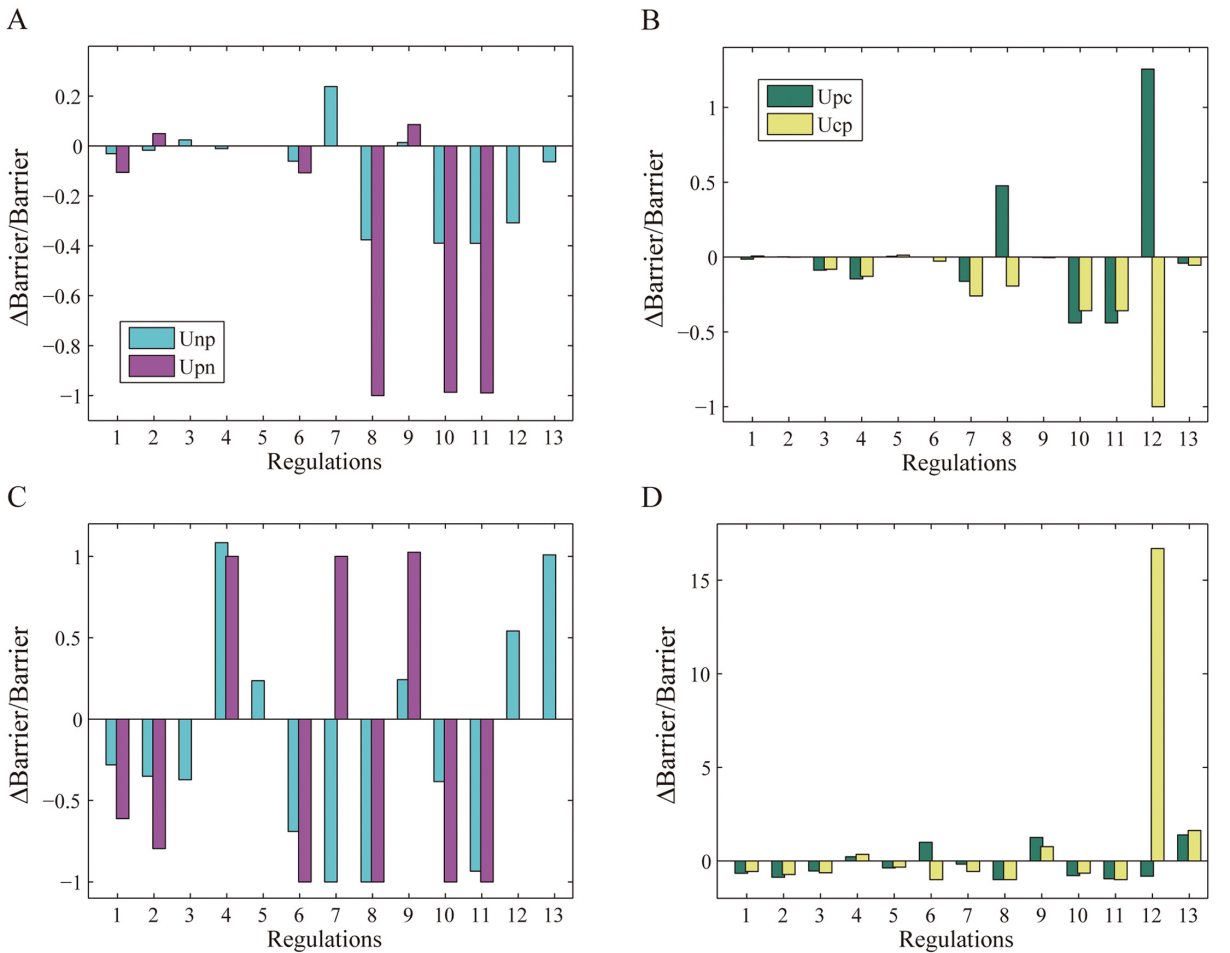
Variation rate of barrier height with regulation strength. The meanings of *U*_*np*_, *U*_*pn*_, *U*_*pc*_ and *U*_*cp*_ are the same as Fig 5.

We further explored the GRN to identify the key genes and regulations crucial for breast cancer formation, prevention and treatment from global sensitivity analysis on landscape topography. In the GRN, each node (gene) and link (regulation) contributes to the breast cancer dynamics. Variation in the regulation strengths will result in changes in the barrier heights between attractor basins. In this way we can recognize genes and regulations that are more sensitive in the GRN, where sensitivity is quantified by the variation rate of barrier height (detailed results are shown in S4 Table). There are two pairs of barrier heights: *U*_*np*_ and *U*_*pn*_; *U*_*pc*_ and *U*_*cp*_. We are interested in cases where the barrier heights in each pair change in opposite directions (e.g., Δ*U*_*np*_ > 0 while Δ*U*_*pn*_ < 0). This corresponds to cell state transformations with a preferred direction and a suppressed reverse direction. For clarity, we vary only one regulation strength each time. In Fig 6 we showed 13 regulations in the GRN, numbered from 1 to 13, which are listed below respectively: (1) E2F1→BRCA1, (2) E2F1→ATM, (3) MDM2→CHEK2, (4) BRCA1→CHEK2, (5) ATR→BRCA1, (6) TP53→ATM, (7) ATM→MDM2, (8) HER2⊣TP53, (9) MDM2⊣TP53, (10) RB⊣E2F1, (11) P21⊣E2F1, (12) CDK2⊣BRCA1, (13) ATR⊣MDM2. Fig 6 (A) and 6(B) show the variation rate of barrier height with the regulation strength reduced to 40% of its original value. Fig 6 (C) and 6(D) show the variation rate of barrier height with the regulation strength doubled and the self-degradation constant of the corresponding node quadrupled at the same time.

We first take a look at Fig 6(B). The 8th (HER2⊣TP53) and 12th (CDK2⊣BRCA1) regulation variation results in a significant increase in *U*_*pc*_ and a significant decrease in *U*_*cp*_. That means it is much more difficult to transform from the premalignant to cancer state and much easier to reverse from the cancer to premalignant state. This kind of landscape topography change is helpful for breast cancer recovery. The effect of the 8th regulation variation can be understood as follows. p53 is the ‘guardian of the genome’ associated with the regulation of DNA repair, cell cycle arrest and apoptosis [35]. When the repression strength of HER2⊣TP53 decreases, the transcription level of p53 increases. Consequently, the functions associated with p53 will be more effective, in agreement with the direction of cancer recovery. On the other hand, reducing the expression level of HER2 has similar effects as decreasing the repression strength of HER2⊣TP53, as in both cases TP53 becomes less repressed. It has been found that HER2 is over-expressed in 18%-20% of invasive breast cancers and about 20% drugs treating breast cancer, such as Trastuzumab and Pertuzumab, are targeted at HER2 by inhibiting its expression level [36, 37]. These popular therapies also agree with the strategy of decreasing the repression strength of HER2⊣TP53 in cancer recovery processes.

In a similar fashion, reducing the repression strength of CDK2⊣BRCA1 makes the reversal from the cancer to premalignant state easier. In contrast, as shown in Fig 6(D), when the repression strength of CDK2⊣BRCA1 is increased (doubled), the change in the barrier heights indicate that it becomes easier to transform from the premalignant to cancer state, while much more difficult to reverse. This is because BRCA1 is a tumor suppressor whose main function is DNA repair. Many researches have shown that the loss of BRCA1 accounts for 80% of breast cancer [38–40]. Therefore, reducing the repression strength of CDK2⊣BRCA1 and enhancing the concentration of BRCA1 is beneficial to breast cancer treatment.

In Fig 6(C), when the activation strength of the 7th regulation (ATM→MDM2) doubled, the barrier height *U*_*np*_ decreased while *U*_*pn*_ increased significantly. That means it is much easier to transform from the normal to premalignant state and much more difficult to the reverse process. This is because as an estrogen receptor alpha (ER-*α*) regulator [41], MDM2 is over-expressed in over 70% ER positive breast cancer. It has also been proposed to be a drug target for cancer treatment [42]. Therefore, as the activation strength of ATM→MDM2 increases, the expression of MDM2 will arise, indicating cancerous development. Correspondingly, as shown in Fig 6(A), reducing the regulation strength of ATM→MDM2 (inhibiting the MDM2 expression) is helpful for preventing normal transforming into premalignant state. The regulation ATM→MDM2 has been studied mostly for DNA damage but not on breast cancer. From the results of our study, more attention should be paid to this regulation due to its importance for breast cancer.

In Fig 6(D), when the activation strength of the 6th regulation (TP53→ATM) increases, the variation rate of the barrier height *U*_*pc*_ increases while that of *U*_*cp*_ decreases. This type of change in the landscape topography contributes to breast cancer recovery, by making it easier to reverse cells in the cancer state to the premalignant state. This has to do with ATM as a chief DNA damage recognition molecule. ATM mutations will cause ataxia-telangiectasia which increases risk of breast cancer. Cell cycle check point mechanisms are weakened during cancerous process as ATM often has a low expression level. Therefore, increasing the activation strength of TP53→ATM and thus the expression level of ATM may assist with breast cancer treatment.

Through global sensitivity analysis, we have thus identified four key regulations (HER2⊣TP53, TP53→ATM, ATM→MDM2, CDK2⊣BRCA1) and six key genes (HER2, TP53, ATM, MDM2, BRCA1 and CDK2). Focusing on the key genes and regulations will offer new insights into anti-cancer drug design of breast cancer by screening specific target genes and key regulations. This will give a guide on designing network-medicine based drugs for breast cancer.

### Phenotypic state switchings and associated variations on key gene expressions

**Fig 7:**
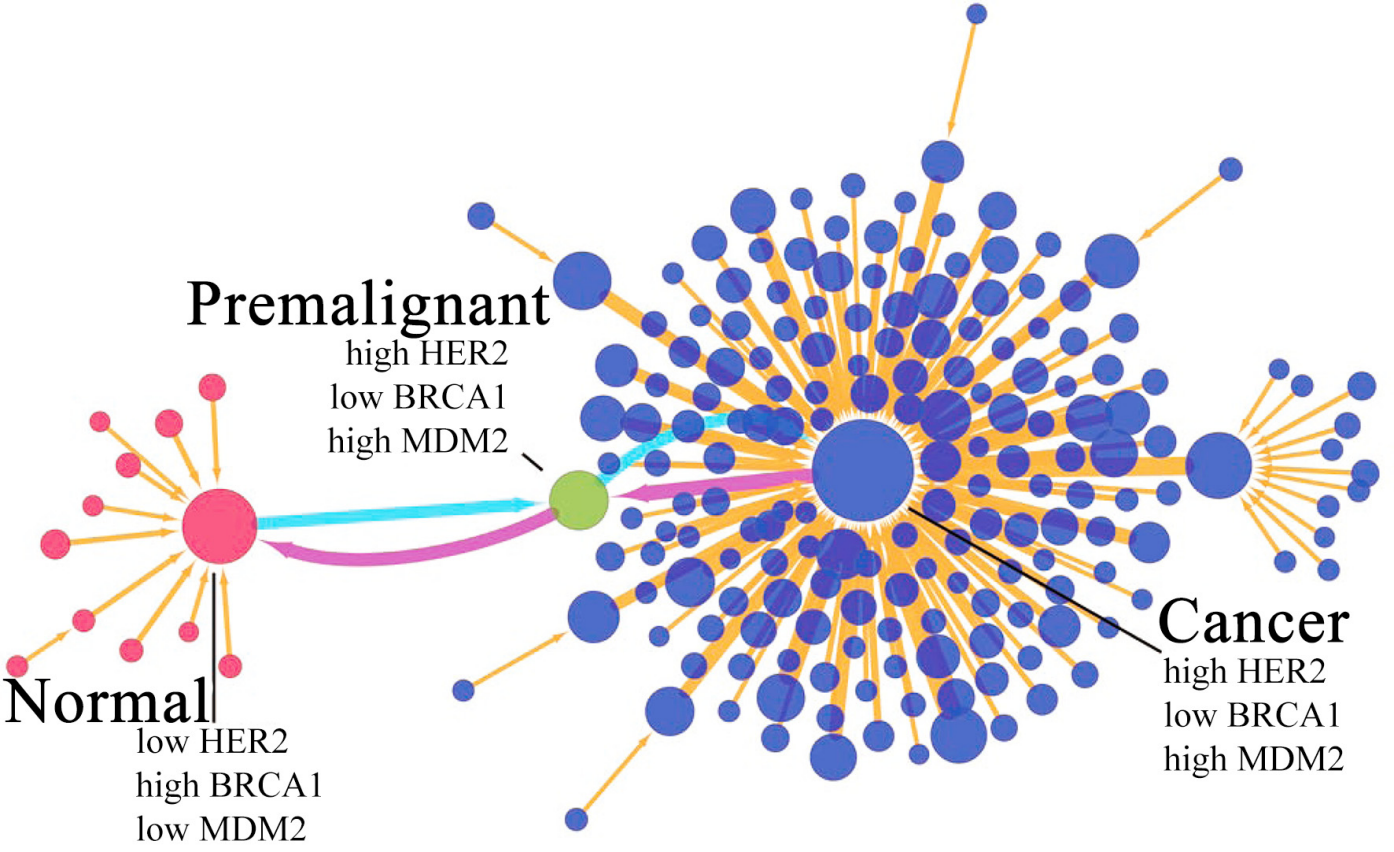
Discrete landscape of the breast cancer GRN. The discrete landscape including 97 nodes (representing the GRN states) and 192 links (representing the transition jumps). The sizes of the nodes and the widths of the links are proportional to the probability of the states and the transition rates, respectively. For clarity of presentation, we set a cut off to discard the nodes with low probabilities and links with small transition rates.

To demonstrate the changes in key gene expression of breast cancer, we draw a discrete landscape. To simplify the representations and high dimensionality for visualization purpose of the results, we use only high and low expression levels to represent the continual expression levels for each gene. There are 2^15^ cell states in the system. The binary code is used to characterized the system: the most significant normal state is (111011100100000) and the most significant cancer state is (000100011011111), where “1” and “0” mean the high expression level and low expression level, respectively. In Fig 7, each node is denoted as a cell state. The expression feature is characterized by three genes: HER2, BRCA1 and MDM2 which are some of the key genes for breast cancer in our global sensitivity analysis results and breast cancer treatment. In the cancer state, the expression level is high in HER2 and MDM2, low in BRCA1, while they are reverse in normal state. In the premalignant state, the gene expression levels are varied but not completely consistent with that in cancer state (see Supplementary S3 Table). The transition jump colored in magenta is made from cancer state to normal state through premalignant state (See Supplementary S5 Table for the state transitions along this path in the discrete state representation). The light blue is along the reverse direction. The changes of the key gene expressions can lead to the system transforming from normal state to cancer state eventually. This is because the variation of protein concentration can result in the cellular environmental change and incorrect signal transduction. Meanwhile, the signal transduction error can also influence the gene expression level in the downstream, which is a vicious circle. As shown in Fig 7, the increase of HER2 and MDM2 expression levels and the decrease of the BRCA1 expression level can drive the system gradually into cancer state. So the detection of premalignant state is important for breast cancer early diagnosis. However, the premalignant state of breast cancer is difficult to identify; the current premalignant breast lesion detection is very limited. We can identify the non-obvious premalignant state by quantifying the landscape topography. This can be realized through a collection of the measurements through the flow cytometry, the quantitative PCR or the microscope measurements via GFP fluorescence labeling. The premalignant state of breast cancer can then be identified at the corresponding peak in the histogram and the associated gene expression levels can be determined. If the expression levels of some vital genes change along the cancer direction, this is an important signal of cancerization. We calculate the threshold for the fifteen genes in our breast cancer network (See Supplementary S3 Table). The threshold value is determined by the position of the barrier in gene expression space between the normal state and premalignant state. We assume if the state has already reached the premalignant state, the system has high likelihood to be in cancer state. Let us take MDM2 for example, MDM2 is over-expressed (high expression level) in breast cancer. If the gene expression level is in the range of lower than 2.4946, it is in a safe area. If the MDM2 gene expression level is in the range of 2.4946 to 3.006222, it is in a warning area. If the gene expression is over 3.006222, it can be said that the gene expression is in a high expression level close to cancer regime, and we should pay more attention to.

The detections of variations of gene expression levels can help us on the early diagnosis and preventions of breast cancer. In premalignant state, TP53, ATM and BRCA1 are at low gene expression levels. MDM2, HER2 and CDK2 are at the high gene expression levels. The amplification of HER2 and MDM2 is an important signal of early diagnosis of breast cancer. Loss of BRCA1 and TP53 is a dangerous warning of breast cancer. The identification of new prognostic markers may provide new ways for the diagnosis and treatments for the breast cancer. Our theoretical results can help in identifying the potential targets for early diagnosis and treatments.

### Landscape topography changes under variations on key gene regulation strengths

**Fig 8:**
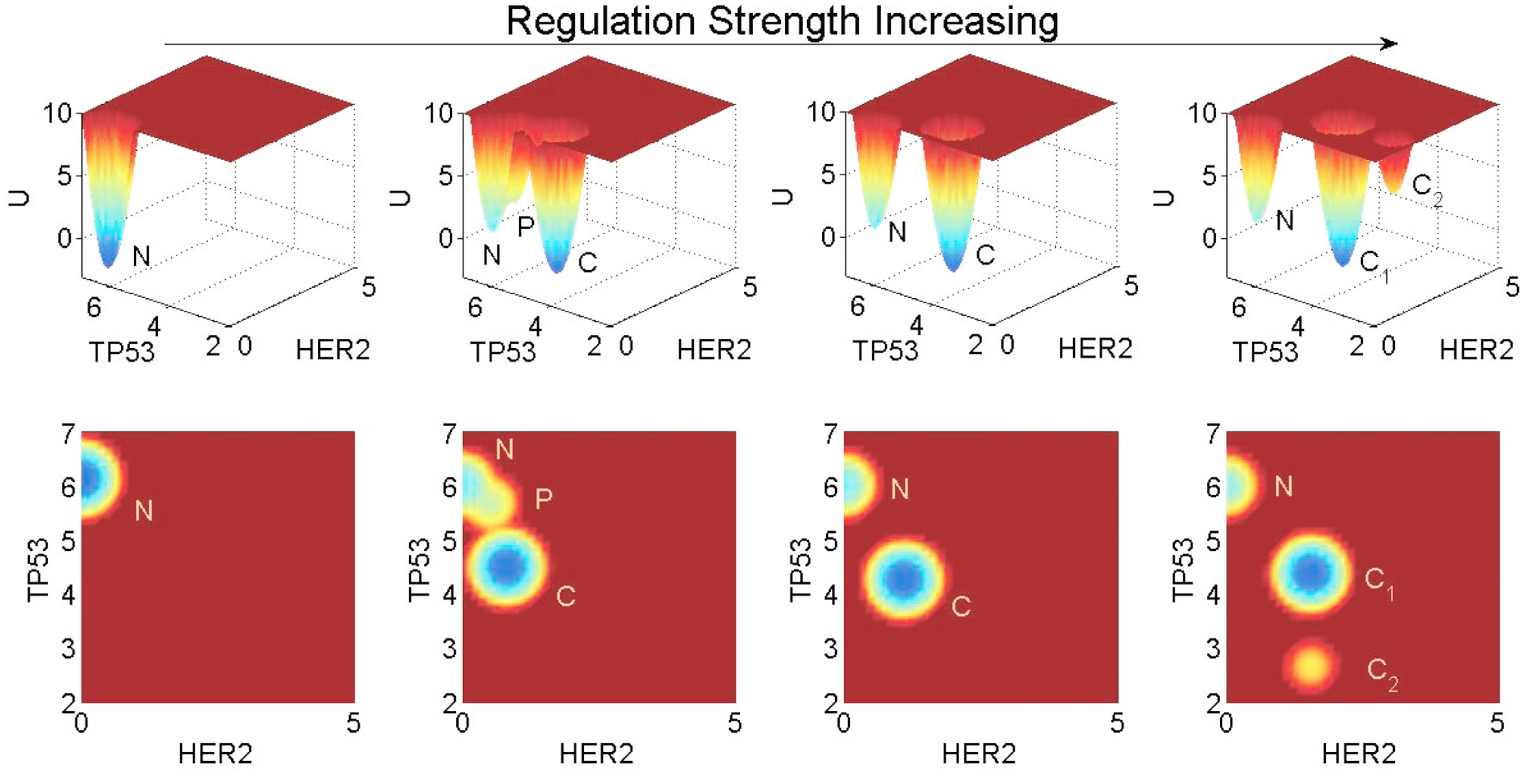
Landscape changes of the GRN as the strengths of the three regulations repressing TP53 vary together. From left to right, the TP53 repression strengths are 60%, 100%, 120%, and 160%, respectively, of the original level.

Genetic mutations and epigenetic factors can affect the gene regulation strengths in the GRN, which in turn has an impact on the landscape topography. To show the influences of key gene regulations on the landscape topography of breast cancer, we display a contrast as Fig 8 when the regulation strengths increasing. In Fig 8 the landscape topography changes as the regulations (HER2⊣TP53, MDM2⊣TP53 and P21⊣TP53) are varied together and others remain the same. The three genes (HER2, MDM2 and P21) are often over-expressed in breast cancer, and both HER2 and MDM2 are the popular drug target of breast cancer at present. If the expression levels of the three genes increasing, the repression of TP53 will increase. When the regulation strengths are reduced to 60%, the normal state is dominant, while the premalignant and cancer states disappear. When the repression strengths are increased to 120%, the cancer state becomes dominant and the premalignant state disappears. When the repression strengths are increased to 160%, another state we named *Cancer*_2_ appears, whose gene expression levels have even more cancerous characteristics. In other words, as the repression strengths increasing, there is a general trend for the system to become more cancerous, as TP53 with a extremely low expression level. This is consistent with our results in global sensitivity analysis. It also agrees with the fact that the over-expression of HER2, MDM2 and P21 will increase the repression of TP53, which result higher risk of breast cancer.

### Conclusion

We constructed a gene regulatory network of breast cancer based on available experimental literatures and database. We then quantified the potential landscape of the GRN. Three biological functional states, namely the normal, premalignant and cancer states, were identified with the corresponding attractors on the landscape. The dynamical transitions between these attractor states were studied with the kinetic paths. We found that the kinetic paths between the normal and premalignant states are almost reversible, while those connecting the premalignant and cancer states are irreversible. We further investigated how the landscape topography is affected by genetic, epigenetic and environmental changes. Through global sensitivity analysis, we identified six key genes (HER2, MDM2, ATM, BRCA1, TP53 and CDK2) and four key regulations (ATM→MDM2, HER2⊣TP53, CDK2⊣BRCA1 and TP53→ATM) for breast cancer.

Our studies indicate that breast cancer is a polygenic disease associated with the formations and transitions of attractors with biological functions on the underlying landscape of the entire gene regulatory network. It is affected by multiple factors including both genetic mutations and non-genetic influences that alter the landscape topography. In particular, increased fluctuations may be an essential component in the mechanism of breast cancer formation. It is therefore optimal to adopt polygenic methods for breast cancer diagnosis, prevention and treatment.

The premalignant state can play a pivotal role in early diagnosis and prevention of breast cancer, as it is less stable than the normal or cancer states and thus relatively easy to transform into them. Yet currently it is still difficult to detect the premalignant state of breast cancer. The detection of premalignant state by testing the variations of gene expression levels is a feasible method for early diagnosis. This way we can fully exploit the potential power of the premalignant state in breast cancer early diagnosis and prevention.

Our global sensitivity analysis shows that changing the strengths of the key regulations in the breast cancer GRN can allow the landscape topography to move in preferred directions that are beneficial for cancer reversion back to normal state. This offers some new insights into the network-medicine based drug design of breast cancer through modulating the key regulation strengths and key expression levels. The six key genes and four key regulations identified in the global sensitivity analysis of our model provide information on genes and regulations to be focused on in the anti-breast-cancer drug design. The modulation of the regulation strengths in our model can serve as quantitative approach in the network-medicine based drug design. Furthermore, among the key regulations, ATM→MDM2 has been studied mostly on DNA damage but not on breast cancer. We suggest the importance of ATM→MDM2 should be noticed on breast cancer study.

The GRN uncovers breast cancer on genetic level including some epigenetic information. For more completely understanding the mechanism of breast cancer, more information should be added into the network, such as metabolic, environmental factors and signal transduction rate etc. to reflect the biological process precisely. In that case, we can acquire more information of breast cancer and go further of this research.

## Materials and Methods

### Self-consistent mean field approximation

For gene regulatory networks, the state of the system can be represented by a vector with n components, **x** = (*x*_1_, *x*_2_, …, *x*_*n*_)^*T*^, where the subscript *T* denotes transpose. *x*_*i*_ (*i* = 1, 2, ⋯, *n*) may represent, for instance, the concentrations of protein species or the expression levels of genes in the model. The deterministic dynamics of the network is described by a set of ordinary differential equations written compactly as $\dot{x}=F\left(x\right)$, where **F**(**x**) is the deterministic driving force.

In cellular environment, intrinsic and external fluctuations cannot be ignore. With fluctuations taken into account, the stochastic dynamics of the system can usually be described by the Langevin equation: $\dot{x}=F\left(x\right)+\xi \left(x,t\right)$, where **ξ**(**x**, *t*) is the stochastic force satisfying 〈**ξ**(**x**, *t*)〉 = 0 and 〈**ξ**(**x**, *t*)**ξ**^*T*^(**x**, *t*′)〉 = 2**D**(**x**)*δ*(*t* − *t*′). The diffusion matrix **D**(**x**) characterizes the fluctuation strength and correlation.

Instead of the following individual stochastic trajectories which are unpredictable, we will follow the probability evolution which is predictable. The temporal evolution of the probability distribution *P*(**x**, *t*) is governed by the corresponding Fokker-Planck (diffusion) equation: ∂*P*/∂*t* = −∇ ⋅ [**F** *P* − ∇ ⋅ (**D** *P*)]. It can be interpreted as a local probability conservation equation ∂*P*/∂*t* = −∇ ⋅ **J**, with probability flux **J** = **F** *P* − ∇ ⋅ (**D** *P*). (For simplicity, consider constant diffusion matrix **D**.) In the steady state, ∂*P*_*ss*_/∂*t* = ∇ ⋅ **J**_*ss*_ = 0. The steady-state probability flux, **J**_*ss*_ = **F** *P*_*ss*_ − **D** ⋅ ∇*P*_*ss*_, when it is deviated from zero, quantifies the degree of the non-equilibrium away from equilibrium. The divergent free nature of the flux ∇ ⋅ **J**_*ss*_ = 0 indicates that the flux is a curl. For non-equilibrium systems, the driving force **F** for the dynamics can be decomposed of a gradient of the potential landscape and a curl flux force [43]: **F** = −**D** · ∇*U* + **J**_*ss*_/*P*_*ss*_, where *U* = −ln *P*_*ss*_ is the potential landscape.

In general, it is difficult to solve the Fokker-Planck (diffusion) equation to obtain the time dependent and the steady state probability/potential landscape. The self-consistent mean field approach [44] provides an approximation by assuming a separable form of the probability distribution *P*(*x*_1_, *x*_2_, ⋯, *x*_*n*_, *t*)∼∏_*i*_ *P*(*x*_*i*_, *t*), so that the probability can be solved self-consistently. The dimensionality in the problem is reduced from *m*^*n*^ to *m* × *n*, making the computation more tractable.

The Gaussian Probability Distribution is used as an additional approximation. For small fluctuations, the mean vector $\bar{x}\left(t\right)$ and covariance matrix **σ**(*t*) of the Gaussian distribution obey the following moment equations:

$$\begin{array}{c} \dot{\bar{x}}\left(t\right)=F\left(\bar{x}\left(t\right)\right) \end{array}$$ (2)

$$\begin{array}{c} \dot{\sigma }\left(t\right)=A\left(t\right)\sigma \left(t\right)+\sigma \left(t\right)A^{T}\left(t\right)+2D\left(\bar{x}\left(t\right)\right). \end{array}$$ (3)

The elements of the matrix **A** are given by $A_{ij}\left(t\right)=\frac{\partial F_{i}\left(\bar{x}\left(t\right)\right)}{\partial {\bar{x}}_{j}\left(t\right)}$. Due to the self-consistent mean field approximation of separable distributions, only diagonal elements of *σ*(*t*) are considered in Eq (3). Thus based on the approximation of separable Gaussian distributions, the evolution of the probability distribution to each variable *x*_*i*_ is given by

$$\begin{array}{c} P\left(x_{i},t\right)=\frac{1}{\sqrt{2\pi \sigma_{i}\left(t\right)}}\exp\{-\frac{{\left[x_{i}-{\bar{x}}_{i}\left(t\right)\right]}^{2}}{2\sigma_{i}\left(t\right)}\}. \end{array}$$ (4)

For a monostable system, the steady-state probability distribution obtained from Eq (4) is a separable Gaussian distribution centered at the fixed point. For a multistable system, there is a separable Gaussian distribution associated with each fixed point. The final steady-state probability distribution *P*_*ss*_(**x**) is constructed as a linear combination of these Gaussian distributions, with the combination coefficients chosen to be the relative frequencies of occurrence of the corresponding fixed points.

### Optimal path through path integral formulation

Consider stochastic systems governed by the Fokker-Planck (diffusion) equation with a constant diffusion matrix: ∂*P*(**x**, *t*)/∂*t* = −∇ ⋅ [**F**(**x**)*P*(**x**, *t*) − **D** ⋅ ∇*P*(**x**, *t*)]. Based on the Onsager-Machlup functional approach [31], the transition probability from the initial state **x**_*ini*_ at time *t*_*i*_ to the final state **x**_*fin*_ at time *t*_*f*_ is given by a path integral: $P\left(x_{fin},t_{f};x_{ini},t_{i}\right)=\int D\left[x\left(t\right)\right]\exp\{-S[x(t)]\}=\int D\left[x\left(t\right)\right]\exp\{-\int L(x(t))dt\}$, where $L\left(x\left(t\right)\right)=\frac{1}{4}\left(\dot{x}-F\left(x\right)\right)\cdot D^{-1}\cdot \left(\dot{x}-F\left(x\right)\right)+\frac{1}{2}\nabla \cdot F\left(x\right)$ is the Lagrangian and S[x(t)]=∫L(x(t))dt is the action. The notation ∫D[x(t)] represents an integral over all the possible paths beginning from the initial state **x**_*ini*_ at time *t*_*i*_ and ending in the final state **x**_*fin*_ at time *t*_*f*_. According to this formula, each path is assigned with a probability weight, exp{−*S*[**x**(*t*)]}, associated with the action of that path. The kinetic paths are identified as the dominant paths with maximum probability. In non-equilibrium systems the non-vanishing curl flux **J**_*ss*_ drives the kinetic path to deviate from the steepest descent path on the landscape. Therefore, the kinetic paths of non-equilibrium systems are in general irreversible.

## Supporting Information

S1 Table Literature search results. These results are mainly from EVEX database. *a* represents activation and *r* represents repression.
(PDF)

S2 Table Gene function of the 15 genes. (PDF)

S3 Table Gene expression value in each state. Gene expression value in each state, the expression values of TP53 and ATM in premalignant state are close to normal state, others are close to cancer state. gene expression characteristic in cancer and normal. “0” represents low expression level, “1” represents high expression level.
(PDF)

S4 Table Global sensitivity analysis calculation results. Table (a) shows the regulation strength is reduced to 40% of its original value. Table (b) shows the regulation strength doubled and the self-degradation constant of the corresponding node quadrupled at the same time. Red tags are used to mark the sensitivity regulations and key genes we have found.
(PDF)

S5 Table Dynamic path. One of the dynamic paths from normal to cancer (11101110010000) is a normal cell state. (111111111011111) is a cancer cell state. The vital genes (MDM2, AKT1, CDK2, E2F1, P21, HER2, RB, RAF, RAS) are gradually evolved to cancer from normal state. This is one of the paths from normal to cancer in discrete manifestation.
(PDF)

S6 Table Expression value in normal state when *a* < 1, *a* = 1, *a* > 1. (PDF)

S1 Fig The flowchart of the algorithm. (TIF)
