# Supplementary material for: A Physical Mechanism and Global Quantification of Breast Cancer
Source: PLoS One. 2016 Jul 13;11(7):e0157422. doi: 10.1371/journal.pone.0157422 (PMC4943646; doi:10.1371/journal.pone.0157422)
Supplement: S1 Table — These results are mainly from EVEX database. a represents activation and r represents repression. (PDF) [file pone.0157422.s001.pdf]

S1 Table: Literature search results.

ATR	TP53	*a*	(1)
ATR	MDM2	*r*	(2)
ATR	BRCA1	*a*	(3)
ATR	CHEK1	*a*	(4)
TP53	ATM	*a*	(5)
TP53	MDM2	*a*	(6)
TP53	E2F1	*r*	(7)
TP53	P21	*r*	(8)
ATM	MDM2	*a*	(9)
ATM	CHEK2	*a*	(10)
MDM2	TP53	*r*	(2)
MDM2	CHEK2	*a*	(11)
BRCA1	ATR	*a*	(12)
BRCA1	TP53	*a*	(13)
BRCA1	CHEK1	*a*	(14)
BRCA1	CHEK2	*a*	(14)
AKT1	TP53	*a*	(15)
AKT1	MDM2	*a*	(16)
AKT1	RAS	*a*	(17)
AKT1	CHEK1	*r*	(18)
AKT1	ATR	*r*	(18)
AKT1	BRCA1	*r*	(19)
CDK2	CDK2	*r*	(20)
CDK2	BRCA1	*r*	(21)
CDK2	ATM	*r*	(22)
E2F1	ATM	*a*	(9)
E2F1	BRCA1	*a*	(23)
E2F1	CHEK2	*a*	(9)
P21	TP53	*r*	(24)

P21	AKT1	*a*	(25)
P21	CDK2	*a*	(20)
P21	E2F1	*r*	(26)
P21	RB	*a*	(27)
P21	RAF	*a*	(28)
HER2	TP53	*r*	(29)
HER2	P21	*a*	(30)
HER2	HER2	*a*	(31)
RB	AKT1	*a*	(32)
RB	E2F1	*r*	(33)
RAS	TP53	*a*	(34)
RAS	RAF	*a*	(35)
