# Supplementary material for: A Physical Mechanism and Global Quantification of Breast Cancer
Source: PLoS One. 2016 Jul 13;11(7):e0157422. doi: 10.1371/journal.pone.0157422 (PMC4943646; doi:10.1371/journal.pone.0157422)
Supplement: S2 Table — (PDF) [file pone.0157422.s002.pdf]

S2 Table: Gene function of the 15 genes.

Gene	Function
ATR	a cell cycle checkpoint gene required for cell cycle arrest and DNA damage repair in response to DNA damage. This kinase has been shown to phosphorylate checkpoint kinase CHEK1,check point protein of tumor suppressor protein BRCA1.
TP53	Tumor suppressor gene
ATM	Similar to gene ATR and helps control the rate at which cells grow and divide. ATM protein assists cells in recognizing damaged or broken DNA strands.
MDM2	Oncogene, recognizes the N-terminal trans-activation domain (TAD) of the p53 tumor suppressor and an inhibitor of p53 transcriptional activation
BRCA1	Oncogene, DNA repair.
CHEK1	Kinase, It is required for checkpoint mediated cell cycle arrest in response to DNA damage or the presence of unreplicated DNA.
CHEK2	Kinase, in response to DNA damage
AKT1	Kinase, helps regulate cell growth and division, control apoptosis
CDK2	Kinase, participate in cell cycle regulation
E2F1	Transcription factor, control of cell cycle and action of tumor suppressor proteins
P21	Tumor suppressor gene
HER2	Oncogene, hormone receptor
RB	Tumor suppressor gene
RAF	Kinase
RAS	Oncogene, regulate cell growth and differentiation
