# Supplementary material for: A Physical Mechanism and Global Quantification of Breast Cancer
Source: PLoS One. 2016 Jul 13;11(7):e0157422. doi: 10.1371/journal.pone.0157422 (PMC4943646; doi:10.1371/journal.pone.0157422)
Supplement: S3 Table — Gene expression value in each state, the expression values of TP53 and ATM in premalignant state are close to normal state, others are close to cancer state. gene expression characteristic in cancer and normal. “0” represents low expression level, “1” represents high expression level. (PDF) [file pone.0157422.s003.pdf]

S3 Table: Gene expression value in each state.

	Gene symbol	Expression in Cancer (mutant)	Expression in Normal (wild type)	literatures	Threshold (P-N)	State1 (cancer)	State2 (premalignant)	State3 (normal)
1	ATR	0	1	(36)	2.0558	1.038881	1.208514	2.993216
2	TP53	0	1	(37)	5.7072	4.501773	5.668179	6.019654
3	ATM	0	1	(38)	1.9904	1.778189	1.983726	1.99748
4	MDM2	1	0	(39)	2.4946	3.148951	3.006222	1.993264
5	BRCA1	0	1	(40)	2.4616	1.889536	2.261368	2.635304
6	CHEK1	0	1	(41)	3.0748	1.93858	2.142378	3.988577
7	CHEK2	0	1	(42)	4.0970	2.997345	3.270013	4.97573
8	AKT1	1	0	(43)	0.5178	1.620486	1.005131	0
9	CDK2	1	0	(44)	0.7466	1.020858	0.845147	0.641891
10	E2F1	0	1	(45)	3.0672	0.761765	1.99111	4.001145
11	P21	1	0	(46)	0.2639	0.811753	0.501372	0.001145
12	HER2	1	0	(47)	0.4444	0.809017	0.5	0
13	RB	1	0	(48)	0.4463	0.810577	0.502055	0
14	RAF	1	0	(49)	0.7646	1.690594	1.351618	0
15	RAS	1	0	(50)	0.4947	0.971463	0.890396	0
