# Supplementary material for: A Physical Mechanism and Global Quantification of Breast Cancer
Source: PLoS One. 2016 Jul 13;11(7):e0157422. doi: 10.1371/journal.pone.0157422 (PMC4943646; doi:10.1371/journal.pone.0157422)
Supplement: S4 Table — Table (a) shows the regulation strength is reduced to 40% of its original value. Table (b) shows the regulation strength doubled and the self-degradation constant of the corresponding node quadrupled at the same time. Red tags are used to mark the sensitivity regulations and key genes we have found. (PDF) [file pone.0157422.s004.pdf]

S4 Table: Global sensitivity analysis calculation results.

(a)

	**Regulation lists**	**△Barrier/Barrier (Unp)**	**△Barrier/Barrier (Upn)**	**△Barrier/Barrier (Upc)**	**△Barrier/Barrier (Ucp)**
1	E2F1->BRCA1	-0.030862	-0.10586	-0.013809	0.0069299
2	E2F1->ATM	-0.016479	-0.049752	0.0014232	-0.0012068
3	MDM2->CHEK2	0.024046	0	-0.087696	-0.08137
4	BRCA1->CHEK2	-0.010009	0	-0.14569	-0.12854
5	ATR->BRCA1	-0.0002537	0	0.0044942	0.013435
6	TP53->ATM	-0.06087	-0.10754	0	-0.027202
7	ATM->MDM2	0.23827	0	-0.16173	-0.25932
8	HER2-|TP53	-0.37622	-1	0.47632	-0.19363
9	MDM2-|TP53	0.014103	0.085795	-0.0022739	-0.0032759
10	RB-|E2F1	-0.38918	-0.98702	-0.43982	-0.35816
11	P21-|E2F1	-0.39053	-0.98936	-0.43976	-0.35815
12	CDK2-|BRCA1	-0.30832	0	1.2554	-1
13	ATR-|MDM2	-0.063283	0	-0.04142	-0.05422

(b)

	**Regulation lists**	**△Barrire/Barrier (Unp)**	**△Barrire/Barrier (Upn)**	**△Barrire/Barrier (Upc)**	**△Barrire/Barrier (Ucp)**
1	E2F1->BRCA1	-0.28013	-0.61203	-0.65941	-0.56538
2	E2F1->ATM	-0.35158	-0.79538	-0.86748	-0.72984
3	MDM2->CHEK2	-0.37241	0	-0.53584	-0.63227
4	BRCA1->CHEK2	1.0848	1	0.21561	0.35558
5	ATR->BRCA1	0.23644	0	-0.37478	-0.33033
6	TP53->ATM	-0.69007	-1	1	-1
7	ATM->MDM2	-1	1	-0.16743	-0.56884
8	HER2-|TP53	-1	-1	-1	-1
9	MDM2-|TP53	0.24202	1.0253	1.2498	0.76277
10	RB-|E2F1	-0.38405	-1	-0.78277	-0.65336
11	P21-|E2F1	-0.93467	-1	-0.95127	-1
12	CDK2-|BRCA1	0.54203	0	-0.81587	16.686
13	ATR-|MDM2	1.0094	0	1.3849	1.6236
