# Supplementary material for: A Physical Mechanism and Global Quantification of Breast Cancer
Source: PLoS One. 2016 Jul 13;11(7):e0157422. doi: 10.1371/journal.pone.0157422 (PMC4943646; doi:10.1371/journal.pone.0157422)
Supplement: S6 Table — (PDF) [file pone.0157422.s006.pdf]

S6 Table: Expression value in normal state when a<1,a=1,a>1

	**Gene symbol**	***a*=0.6** (normal)	***a*=0.8** (normal)	***a*=1** (normal)	***a*=1.2** (normal)	***a*=1.4** (normal)
1	ATR	2.588130	2.791151	2.993216	3.194658	3.395694
2	TP53	5.327534	5.647219	6.019654	6.408080	6.802589
3	ATM	1.198336	1.597887	1.99748	2.397093	2.796717
4	MDM2	1.173189	1.587094	1.993264	2.396276	2.797854
5	BRCA1	1.836427	2.235760	2.635304	3.034970	3.434708
6	CHEK1	3.183835	3.586578	3.988577	4.390075	4.791239
7	CHEK2	3.743852	4.365337	4.97573	5.581517	6.185022
8	AKT1	0	0	0	0	0
9	CDK2	0.641891	0.641891	0.641891	0.641891	0.641891
10	E2F1	4.001652	4.001387	4.001145	4.000950	4.000794
11	P21	0.001652	0.001387	0.001145	0.000950	0.000794
12	HER2	0	0	0	0	0
13	RB	0	0	0	0	0
14	RAF	0	0	0	0	0
15	RAS	0	0	0	0	0
