# Supplementary figures and images for: A Physical Mechanism and Global Quantification of Breast Cancer
Source: PLoS One. 2016 Jul 13;11(7):e0157422. doi: 10.1371/journal.pone.0157422 (PMC4943646; doi:10.1371/journal.pone.0157422)

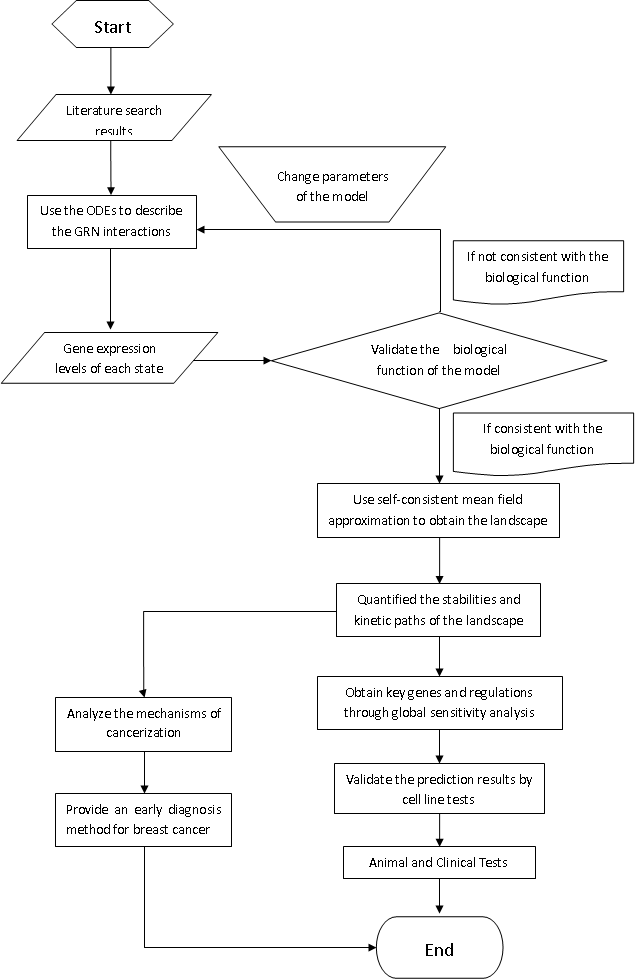

Supplement: S1 Fig — (TIF) [file pone.0157422.s007.tif]
